# Zebrafish: An In Vivo Screening Model to Study Ocular Phenotypes

**DOI:** 10.1167/tvst.11.3.17

**Published:** 2022-03-14

**Authors:** Wim H. Quint, Kirke C. D. Tadema, Johan H. C. Crins, Nina C. C. J. Kokke, Magda A. Meester-Smoor, Rob Willemsen, Caroline C. W. Klaver, Adriana I. Iglesias

**Affiliations:** 1 Department of Ophthalmology, Erasmus Medical Center, Rotterdam, the Netherlands; 2 Department of Clinical Genetics, Erasmus Medical Center, Rotterdam, the Netherlands; 3 Department of Epidemiology, Erasmus Medical Center, Rotterdam, the Netherlands; 4 Department of Ophthalmology, Radboud University Medical Center, Nijmegen, the Netherlands; 5 Institute of Molecular and Clinical Ophthalmology Basel, Basel, Switzerland

**Keywords:** refractive error, FBN1, PRSS56, Marfan syndrome, nanophthalmos

## Abstract

**Purpose:**

To establish a set of assays that allow the in vivo screening of candidate genes for ocular diseases in zebrafish, with an emphasis on refractive error.

**Methods:**

Our pipeline includes the most relevant ocular screening measurements to assess (1) ocular biometry using spectral domain optical coherence tomography, (2) refractive status using an eccentric photorefractor, (3) intraocular pressure by tonometry, and (4) optokinetic response to study visual capability in zebrafish. To validate our pipeline and to demonstrate the potential of zebrafish as a valid animal model, we chose two well-characterized genes with an ocular phenotype (*PRSS56* and *FBN1*) and generated two mutant zebrafish lines (*prss56* and *fbn1*). Mutant fish were assessed at 2, 4, and 6 months after fertilization.

**Results:**

With the proposed phenotyping pipeline, we showed that ocular biometry, refractive status, intraocular pressure, and visual function can be studied in zebrafish. In the *prss56* mutant, the pipeline revealed a dramatic decrease in axial length, mainly owing to a decreased vitreous chamber depth, whereas in the *fbn1* mutant, ectopia lentis was the most distinctive ocular phenotype observed. Tonometry in both mutant lines showed an increase in intraocular pressure.

**Conclusions:**

The proposed pipeline was applied successfully in zebrafish and can be used for future genetic screenings of candidate genes. While validating our pipeline, we found a close resemblance between the ocular manifestations in the zebrafish mutants and patients harboring mutations in *PRSS56* and *FBN1*. Our results support the validity of our pipeline and highlight the potential of zebrafish as an animal model for in vivo screening of candidate genes for ocular diseases.

## Introduction

The development of highly efficient genome editing tools such as CRISPR/Cas9 has enhanced the generation of animal models. Rapid and efficient generation of animal models is essential for functional validation of candidate genes and causal variants derived from genetic studies, including genome-wide association studies. Higher vertebrates varying from rodents to primates have been often used; however, generating stable and representative animal models was often challenging and time consuming. One of the disciplines in which genetic studies have produced a plethora of new candidate genes is ophthalmology. This asks for rapid and efficient functional genetic screening of these genes, such as for myopia and refractive errors. Almost 500 loci have now been identified for these traits, but it remains unclear whether the annotated genes in these loci are involved in their development.^1^^,^^2^

Zebrafish (*Danio rerio*) are increasingly used as a biological model system. Although evolutionary further from humans, they have significant advantages as an in vivo animal model for ocular disease when compared with more traditionally used higher vertebrates, such as rodents. Most rodents have rod-dominant vision, whereas zebrafish, like humans, have cone-dominant vision.^3^^,^^4^ Another advantage of zebrafish is the rapid eye development starting as early as 24 hours after fertilization^4^ to become fully functional at 5 days after fertilization.^4^^–^^6^ This process is significantly faster than murine and avian models, in which ocular development can only be monitored from birth. Additionally, zebrafish are relatively easy and inexpensive to breed with their ability to produce at least 300 eggs per week. As a result, the duration and costs for the creation of a stable mutant using CRISPR/Cas9 technology decreases considerably.

In this study, we described a battery of phenotypic assays that can be used to characterize zebrafish ocular function and pathology, with a particular emphasis on refractive error. To validate our phenotyping pipeline, we chose two well-known genes, Serine Protease 56 (*PRSS56*) and Fibrillin-1 (*FBN1*). *PRSS56* encodes a serine protease involved in eye development, whereas *FBN1* encodes a preprotein, Profibrillin, that is processed into the extracellular matrix glycoprotein as Fibrillin-1 and the protein hormone Asprosin.^7^^,^^8^ Mutations in *PRSS56* cause nanophthalmos or autosomal-recessive posterior microphthalmos^9^^–^^13^; mutations in *FBN1* have been linked to Marfan syndrome (MFS) and Weill–Marchesani syndrome, both of which are characterized by high myopia and ectopia lentis.^14^^–^^17^ Furthermore, common genetic variants in these two genes have also been associated with myopia and refractive error in genome-wide association studies studies,^1^^,^^2^^,^^18^^,^^19^ that is, an exonic variant in *PRSS56* (rs1550094-A, direction hyperopia) and an intronic variant in *FBN1* (rs34539187-C, direction myopia). In the current study, we adapted commonly used techniques for the in vivo testing of ocular biometry, refractive error, and intraocular pressure (IOP) for application in zebrafish. We then evaluated whether depletion of zebrafish *prss56* and *fbn1* resembles the ocular manifestations described in patients and in mouse models carrying mutations in these genes. Using this pipeline, we show the potential of zebrafish to study functional genetics.

## Methods

### Fish Lines and Housing

The mutant lines were generated using CRIPSR-cas9 in wild-type (WT) AB zebrafish. Guide RNAs were designed for *prss56* (TGCTGTAGATGCTGCCGTATCGG; chr2:45196347-45196369; exon 5) and *fbn1* (GATGCAGGTGTAGTTTCCTATGG [reverse complement]; chr18:5517889-555178911; exon 9; downloaded from Ensembl Release 91: December 2017). Frame shift mutations led to premature STOP codons. In *prss56,* a 5bp deletion (-CGTAT) was introduced in exon 5 at 689bp (long isoform: XM_017352217.2) and at 598bp (short isoform: XM_017352214.2). In *fbn1*, we introduced a 1-bp deletion in exon 9 at 5325bp (XM_017351990.2) and an insertion of 5bp (+CTACC). The mutations are predicted to lead to truncated Prss56 (p.(Val24GlyfsTer5)) and Fbn1 (p.(Gly1709AlafsTer40)) proteins. Induced mutations were validated by Sanger sequencing. The mutant lines were registered at the Zebrafish Information Network and line names were assigned (*prss56^re11^* and *fbn1^re12^*) following Zebrafish Information Network nomenclature.

Experimental lines were raised in tanks with matched population sizes and constant feeding patterns, to minimize the environmental influence on ocular development. During spectral domain optical coherence tomography (SD-OCT), photorefraction, IOP, and optokinetic response (OKR) measurements, zebrafish were anesthetized using a 0.016% tricaine methanesulfonate solution (MS222, Sigma Aldrich), buffered to a pH of  7. All animals were treated in accordance to the Dutch animal welfare legislation and the guidelines from the experimental animal health care center (EDC: Experimenteel Dier Centrum) of the Erasmus Medical Center Rotterdam, the Netherlands, and in accordance with the European Commission Council Directive 2010/63/EU (CCD approval, license AVD 1010020186907), and conformed to the ARVO Statement for the Use of Animals in Ophthalmic and Vision Research. All fish were kept on a 14-hour light:10-hour dark cycle at a constant temperature of 28.5°C.

### RNA Isolation and Expression Analysis of *prss56* and *fbn1* in Zebrafish Eyes

Enucleated 6 months postfertilization (mpf) fish eyes were collected (*n* = 4 eyes) for each line and lenses were removed under a stereomicroscope (Leica M80). The tissue was collected in a 1.5 mL Eppendorf tube and immediately frozen in liquid nitrogen until further processing. To extract RNA, 500 µL of Trizol reagent (Ambion, Inc, Austin, TX) was added to the frozen tissue. This mixture was then homogenized using a handheld homogenizer (Pro 200, Pro Scientific Inc., Oxford, CT) for 2 × 5 seconds. The homogenate was left at room temperature for 5 minutes before 200 µL of chloroform (≥99%; Sigma Aldrich, St Louis, MO) was added. The samples where then incubated at room temperature for 15 minutes and centrifuged at maximum RPM for 15 minutes at 4°C. Next, the aqueous fraction was collected and RNA was isolated using the RNeasy micro kit (Qiagen, Hilden, Germany). The extracted RNA was quantified using a Nanodrop (DS-11 Series Spectrophotometer/fluorometer; DeNovix, Wilmington, DE) and cDNA was synthesized using the iScript cDNA Synthesis kit (BioRad, Hercules, CA).

The presence of *prss56* and *fbn1* cDNA transcripts was confirmed by polymerase chain reaction, using the primer sets described in Supplementary Table S1. The amplicons were loaded on agarose gel to confirm expression. To determine the presence of the two predicted isoforms of *prss56*, XM_017352214.2 (short isoform) and XM_017352217.2 (long isoform), the two products of primer set 1 (Supplementary Table S1) were isolated from the agarose gel and re-amplified by polymerase chain reaction. These amplicons were sequenced to identify the two *prss56* isoforms. Protein and DNA alignments were analyzed using Clustal Omega (EMBL-EBI) and Jalview.^20^ For the generation of the phylogenetic tree in Supplementary Figure S2, the alignment data was imported into Geneious prime (Geneious 2020.2), here an UPGMA tree was generated using the Tamura-Nei model and was bootstrapped 100 times.

### Spectral Domain Optical Coherence Tomography

**Figure 1.**
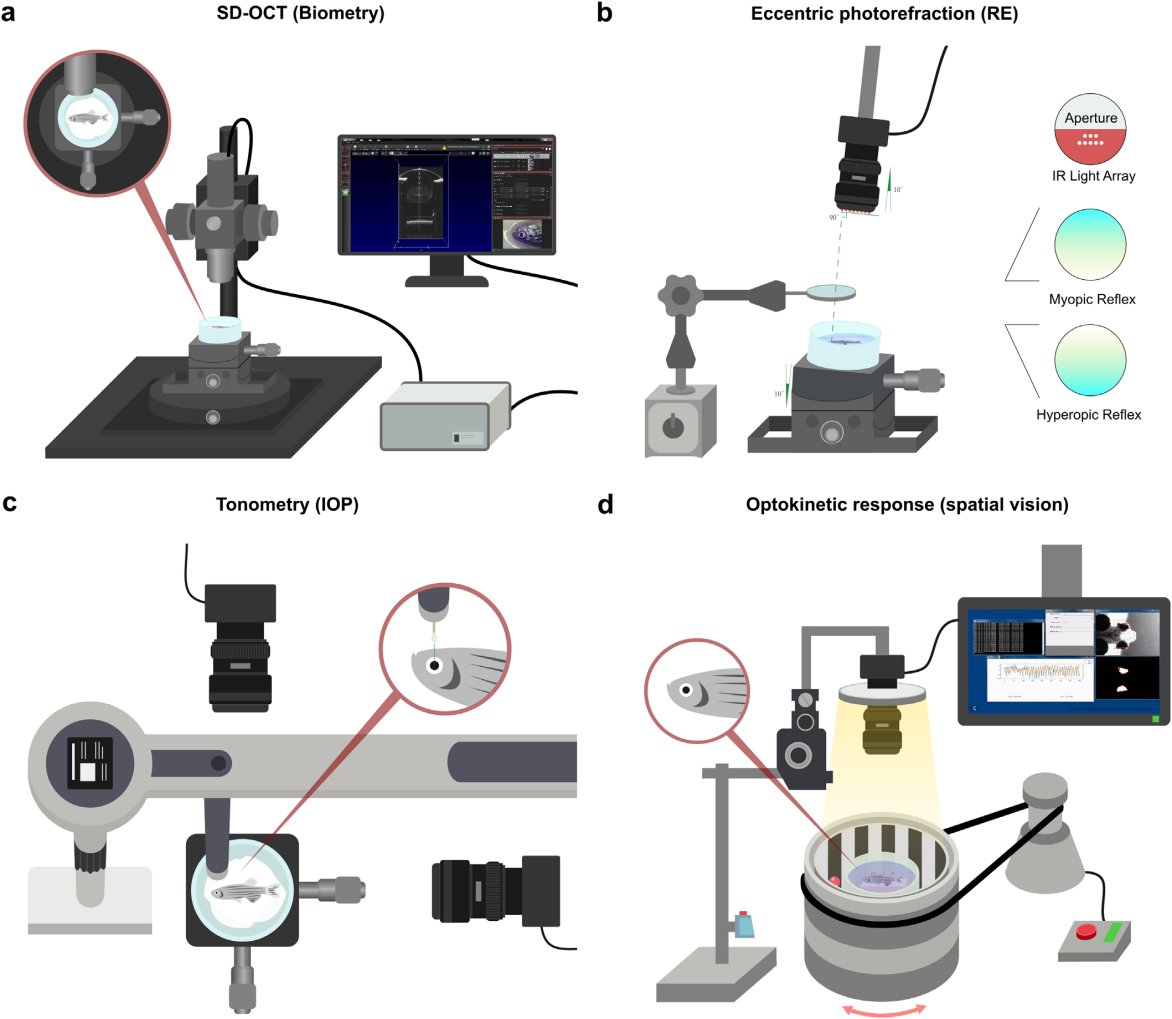
Human ophthalmic assays can be applied in zebrafish to measure ocular performance. Schematic overview of the pipeline of assays used to screen for ocular phenotypes in zebrafish. (a) SD-OCT to assess ocular biometry. (b) Eccentric photorefraction to measure refractive error. (c) Rebound tonometry to measure IOP. (d) The OKR to study visual capability. For full protocols see Supplementary Materials.

The SD-OCT measurements and analysis were performed as described in Figure 1a and Supplementary Materials (Protocol 1). Measurements were performed in WT fish, *prss56* mutants, and *fbn1* mutants fish at 2, 4, and 6 mpf. At these time points, 20 eyes (10 fish), of each genotype were measured. To rule out variability in eye metrics owing to alterations in body length, the mean body length of mutant fish used in this study had a maximum size difference of 1% relative to WT control fish (Supplementary Fig. S1). A Thorlabs SD-OCT 900 nm Ganymede system was used to make three-dimensional scans with a total field of view of 1.7 × 1.7 × 2.2 mm and a pixel depth of 2 µm in the Z-direction. A custom MATLAB script was used for the analysis of the ocular components and total axial length, as described previously.^21^ The dimensions of the ocular components were corrected for the refractive index of each ocular component: the cornea 1.33,^22^^–^^24^ the lens (gradient refractive index) 1.40,^22^^–^^27^ the anterior and vitreous chamber 1.34, and the retina 1.38.^24^^,^^28^^,^^29^ Examiners were not masked to the genotype of the fish. However, image acquisition and manual segmentation were performed independently.

### Eccentric Photorefraction and Relative Refractive Error

The refractive state of the zebrafish eyes was determined by a custom eccentric infrared photorefractor and analyzed by custom software written in C++ (Fig. 1b and Supplementary Materials). WT, *prss56*, and *fbn1* mutant fish were measured at 2, 4, and 6 mpf. There were 100 independent measurements taken for each eye and averaged, a total of 20 eyes (10 fish) per genotype were examined. The slope of the brightness gradient was converted into refractive error by calibrating the system with ophthalmic lenses as described elsewhere.^21^ Further details of this protocol can be found in Supplementary Materials (Protocol 2).

The relative refractive error was computed as described previously.^30^ The relative refractive error was calculated as 1 – (retinal radius/idealized focal length). Here, the idealized focal length is based on a logistic regression of the lens radius vs retinal radius of WT fish from 2, 4, and 6 mpf (*n* = 60 eyes), leading to the formula: idealized focal length = lens radius × 2.182 + 11.699. Relative refractive error values that are lower than zero indicate myopia, whereas values of greater than zero indicate hyperopia.^30^

### IOP Measurements

The IOP was measured using a rebound tonometer (iCare, Vantaa, Finland) fixed to a movable stage (Fig. 1c and Supplementary Materials). Anesthetized fish were positioned with the center of the cornea aligning with the center of the probe. The correct positioning of the probe relative to the cornea during the momentary contact with the eye was monitored and magnified by a perpendicularly positioned USB camera (RICOH, TV LENS 50 mm 1:1,4). For each genotype 10 eyes (five fish) were measured at 2, 3, 6, and 9 mpf. Six measurements for each eye were averaged (see Supplementary Video File S1). Further details of this protocol can be found in Supplementary Materials (Protocol 3).

### Visual Capability Assessment

Visual capability measurements based on spatial performance were measured by monitoring the OKR. OKR assessment was performed using a previously described custom setup^21^ consisting of: a computer, a backlight (TCAM, Ring Light, 0%–100%/12V/6000–7000K), an infrared-emitting diode (XIASONGXIN LIGHT, 9–12 V/10 W/1050 MA), a camera (Ricoh, TV lens 50 mm 1:1,4), a tachometer (Autoleader, NJK-5002C), an electrical motor (Makeblock, 37 MM/DC 12.0 V/50 RPM ± 12%/1:90), and an 850-nm long-pass filter (Midopt, LP695-46). The contrast of the black and white pattern was kept at 100%, the drum velocity at 20 degrees/second, and the spatial frequency at a baseline level of 0.15 cycles/degree. The eyes were tracked in real time by custom-developed software written in Python. Fish were first anesthetized and fixated dorsally on a platform inside a cylindrical and transparent water tank. After this fixation step, the fish were held in fresh system water for 5 minutes to recover from the anesthesia and prestimulated for 5 seconds in both directions. Eye movements were measured binocularly and multidirectional. The OKR graphs were analyzed in Python (version 3.8.0). The slope of the slow phase of the OKR pattern was quantified resulting in the eye velocity in degrees per second. In total 10 fish were measured for each genotype. The values of the temporally or nasally directed eye movements were averaged for each fish. The optokinetic gain was computed as the ratio between the slow phase eye-tracking velocity and the target velocity. This process resulted in a total of 10 data points for each direction. Additionally, the number of eye tracking movements (ETMs) per 15-second interval were quantified during a clockwise drum rotation. During this unidirectional movement, the fish eyes moved binocularly and the number of ETMs was equal for both eyes. For more information see Figure 1d, Supplementary Materials (Protocol 4), and Supplementary Video File S2. The custom Python scripts and corresponding protocols are available on request.

### Statistical Analysis

We analyzed the SD-OCT, photorefraction, and IOP data by fitting a linear mixed model as implemented in GraphPad Prism 8.0. This mixed model uses a compound symmetry covariance matrix, and is fit using restricted maximum likelihood. With this model, both eyes of the same fish are treated as repeated measures. The optokinetic gain data was analyzed by averaging the temporal to nasal eye movements and the nasal to temporal eye movements for each fish, whereas the number of ETMs per 15-second interval were unidirectionally measured during a clockwise drum orientation. The OKR data, as well as the body length measurements, were analyzed by Welch's analysis of variance in GraphPad Prism 8.0.

### Sample Size Calculation

To estimate the sample size for the SD-OCT and photorefraction assessment, we used the GLIMMPSE software^31^ for repeated measures. Estimated effect sizes (i.e., approximately 4% change in axial length and approximately 6% change in photorefraction) were based on a previous study,^21^ alpha-error was set to 0.05 and power to 0.95. Based on these parameters, a minimum size of 20 eyes (10 fish) per group was required (power = 0.953). To calculate the sample size for the OKR we used the G*Power software.^32^ Given an effect size of 1.15 (approximately a 25% decrease in optokinetic gain) a minimum sample size of 10 measurements (10 fish) was estimated. Regarding the IOP, no preliminary data using rebound tonometry in zebrafish were available. The IOP was measured in a subset of 10 eyes (5 fish) per group, with this sample size we estimated, using the GLIMMPSE software,^31^ a power of 0.885 to detect approximately 4.3 mm Hg changes in the IOP caused by genotype at a *P* value of less than 0.05.

## Results

Figure 1 depicts the assays in our pipeline, which includes an assessment of ocular biometry, photorefraction, IOP, and OKR. These assays were used to study the ocular phenotypes of two mutant zebrafish lines, *prss56* and *fbn1*, for which extensive ocular phenotyping has been performed in mouse models but not in zebrafish.^9^^,^^11^^,^^33^^–^^35^ Full step-wise protocols for each technique can be find in the Supplementary Materials.

### *Prss56* and *fbn1* are Expressed in the Zebrafish Eye

**Figure 2.**
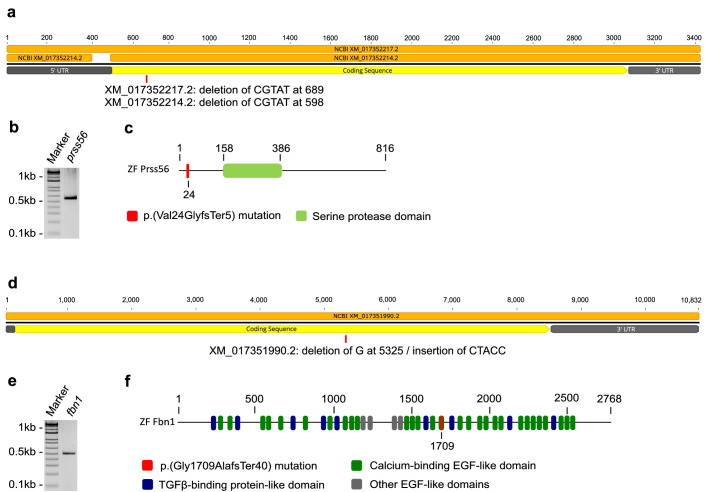
Expression of *prss56* and *fbn1* and characteristics of the induced mutations. Schematic overview of predicted cDNA and protein structures and induced mutations in mutant zebrafish. (a) Schematic illustration of the cDNA structure of *prss56*. The two isoforms XM_017352214.2 and XM_017352217.2 are depicted in orange and the induced mutations are shown for each isoform at the approximate location. (b) Agarose gel loaded with reverse transcriptase polymerase chain reaction product (primer set 4) of isolated RNA extracted from 6 mpf zebrafish eyes, confirming ocular expression of *prss56*. See Supplementary Table S1 and Supplementary Fig. S3 for more information on primers. (c) Schematic illustration of the Prss56 protein structure and induced mutation (in red). (d) Schematic illustration of the cDNA structure of *fbn1*. (e) Agarose gel loaded with reverse transcriptase polymerase chain reaction product of isolated RNA extracted from 6 mpf zebrafish eyes, confirming ocular expression of *fbn1*. See Supplementary Table S1 for primers. (f) Schematic illustration of the Fbn1 protein structure and induced mutation (in red).

We first confirmed the expression of *prss56* and *fbn1* in the zebrafish eye and used CRISPR-cas9 to generate knockout (KO) fish lines. The predicted cDNA and protein structures of both genes are illustrated in Figure 2. Our RT-polymerase chain reaction shows that both *prss56* and *fbn1* are expressed in the zebrafish eye (Figs. 2b, e, Supplementary Fig. S3). NCBI predicted two isoforms for *prss56* (Fig. 2a). Further alignment assessment showed that the differences between both isoforms were limited to the 5’UTR region and are, therefore, not expected to result in structural and functional differences for the Prss56 protein. For further details see Supplementary Materials and Supplementary Figure S3.

With CRISPR/Cas9, we introduced frame shift mutations in both genes resulting in premature stop codons. For *prss56* we introduced a 5-bp deletion (CGTAT) in exon 5 at 689bp for XM_017352217.2 and 598bp for XM_017352214.2. The predicted p.(Val24GlyfsTer5) change in Prss56 protein, prevents the translation of the serine protease domain (Fig. 2c). For *fbn1* we introduced a 1bp deletion (G) in exon 9 at 5325bp (XM_017351990.2) and an insertion of 5bp (CTACC), predicted to result in a p.(Gly1709AlafsTer40) change in Fbn1 protein (Fig. 2f).

### Distinctive Phenotypes for *prss56* and *fbn1* Mutants Detected by SD-OCT

We measured the ocular biometry for the *prss56* and *fbn1* mutants with SD-OCT (for details see Supplementary Table S2). To isolate the intraocular from the extraocular biometrical changes we included fish that show a comparable body length, i.e., max 1% different from the mean body length of WT-fish (Supplementary Fig. S1).

**Figure 3.**
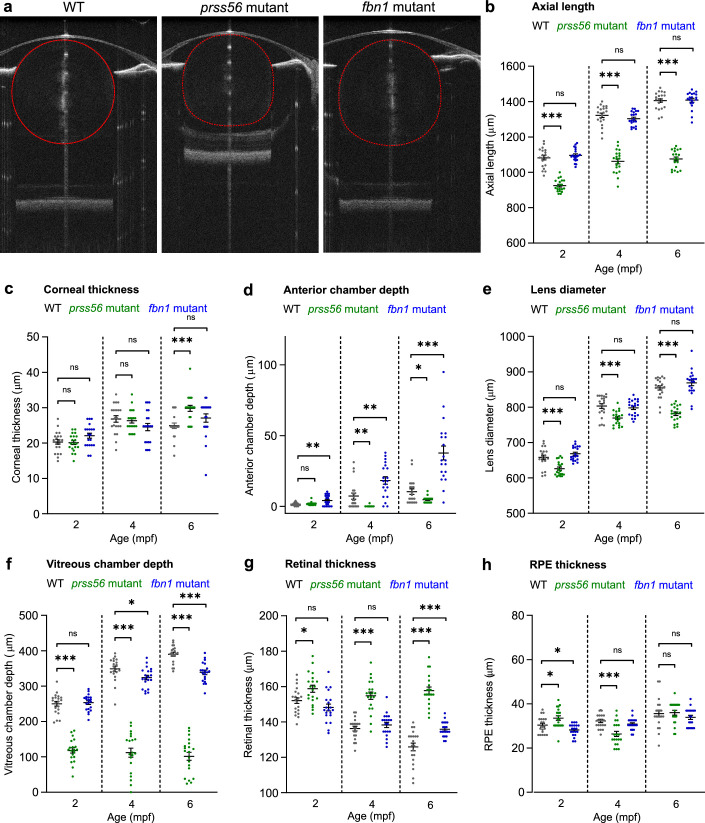
Loss of *prss56* or *fbn1* leads to distinctive biometrical changes in the eye. SD-OCT recordings of size-matched 2 to 6 mpf zebrafish biometrics of ocular compartments. Individual metrics of each compartment of the eye were corrected for the tissue-specific refractive index. (a) A single B-scan image of a typical 4 mpf zebrafish eye of respectively a WT, *prss56* mutant, and *fbn1* mutant. (b) Eyes of the *prss56* mutants were significantly reduced in axial length compared with WT eyes at 2 mpf (effect size = −157 µm; *P* < 0.001), 4 mpf (effect size = −260 µm; *P* < .001), and 6 mpf (effect size = −330 µm; *P* < 0.001). The *fbn1* mutant eyes showed no significant alteration relative to WT. (c) The corneal thickness of 6 mpf *prss56* mutants was significantly increased (effect size = 5 µm; *P* < 0.001). No significant changes in corneal thickness were found in the *fbn1* mutants. d The ACD in the *prss56* mutants was significantly decreased at 4 mpf (Effect size = −7 µm; *P* < 0.01) and 6 mpf (Effect size = −6 µm; *P* < 0.05). The ACD was significantly increased in the *fbn1* mutants at 2 mpf (effect size = 3 µm; *P* < 0.05), 4 mpf (effect size = 11 µm; *P* < 0.01), and 6 mpf (effect size = 27 µm; *P* < 0.001). (e) The lens diameter was significantly reduced in the *prss56* mutants at 2 mpf (Effect size = −32 µm; *P* < 0.05), 4 mpf (effect size = −34 µm; *P* < 0.01), and 6 mpf (effect size = −73 µm; *P* < 0.001). The *fbn1* mutant eyes showed no significant alteration in lens diameter relative to WT. f The VCD of the *prss56* mutants was significantly reduced at 2 mpf (effect size = −132 µm; *P* < 0.001), 4 mpf (effect size = −237 µm; *P* < 0.001), and 6 mpf (effect size = −289 µm; *P* < 0.001). The *fbn1* mutants showed a decrease in VCD at 6 mpf (effect size = −51 µm; *P* < 0.001). (g) The retinal thickness was larger in *prss56* mutants at 2 mpf (effect size = 7 µm; *P* < 0.05), 4 mpf (effect size = 19 µm; *P* < 0.001), and 6 mpf (effect size = 32 µm; *P* < 0.001) and remained stable (range, 155–160 µm), whereas the WT retina is thinning over time (152 µm at 2 mpf; 137 µm at 4 mpf; 126 µm at 6 mpf). The retinal thickness was relatively increased in 6 mpf *fbn1* mutants (effect size = 10 µm; *P* < 0.01). (h) The RPE thickness was significantly altered in 2 mpf (effect size = 3 µm; *P* < 0.05) and 4 mpf (effect size = −5 µm; *P* < 0.001) *prss56* mutants while no significant differences were found in the *fbn1* mutants. Sample size: *n* = 20 eyes for each genotype and time point. See Supplementary Table S2 for all statistics. Error bars: standard error of the mean. Significance: ns = not significant, *P  <  0.05, **P  <  0.01, ***P < 0.001. Scale bar, 100 µm. Mpf, months postfertilization; ACD, anterior chamber depth; VCD, vitreous chamber depth; RPE, retina pigmented epithelium.

The loss of *prss56* led to a decrease in eye volume (Fig. 3a) relative to WT control fish. The total axial length was significantly decreased at 2, 4, and 6 mpf (Fig. 3b), as a result of a decrease in the vitreous chamber depth (Fig. 3f) and lens diameter (Fig. 3e). In the *prss56* mutant, the retinal thickness remained constant (range, 155–160 µm) between 2 and 6 mpf, whereas the retinal thickness of the WT fish decreased over time (152 µm at 2 mpf, 137 µm at 4 mpf, and 126 µm at 6 mpf) (Fig. 3g). This inverse relationship between retinal thickness and globe volume is part of normal eye development and has been reported in previous studies.^36^^–^^39^ *Prss56* mutant showed an increase in corneal thickness at 6 mpf (Fig. 3c), and the anterior chamber depth was decreased at 4 and 6 mpf (Fig. 3d). The retinal pigment epithelium thickness was slightly increased at 2 mpf, whereas it decreased at 4 mpf (Fig. 3h).

The loss of *fbn1* led to a posterior lens dislocation (Fig. 3a); the total axial length was not significantly altered at 2, 4, and 6 mpf (Fig. 3b). Owing to the posterior shift of the lens, the depth of the anterior chamber was significantly increased at all studied time points (Fig. 3d) and the vitreous chamber was significantly decreased at 6 mpf (Fig. 3f). At 6 mpf, the retinal thickness of the *fbn1* mutants was relatively enlarged compared with WT fish (Fig. 3g).

### Mutant *prss56* and *fbn1* Fish Show Altered Refractive State, IOP, and Visual Capability

**Figure 4.**
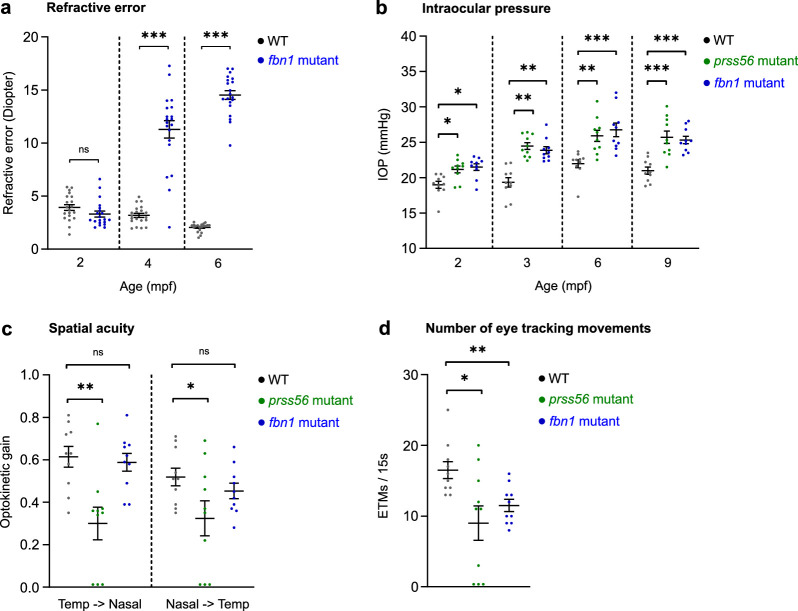
*Prss56* and *fbn1* mutants show an altered refractive status, IOP, and visual capability. Measurements of refractive error, IOP, and the capability for spatial vision. (a) The loss of *fbn1* resulted into a hyperopic shift of the refractive status at 4 mpf (effect size = +8D; *P* < 0.001) and 6 mpf (effect size = +12D; *P* < 0.001), relative to WT (*n* = 20 eyes per timepoint). (b) IOP measured by rebound tonometer (*n* = 10 eyes per timepoint). *Prss56* and *fbn1* mutants both showed significantly increased IOP values relative to WT at 2, 3, 6, and 9 mpf. (c, d) OKRs measured in 6 mpf *prss56* and *fbn1* mutants (*n* = 10 fish). An *n* = 3 *prss56* mutants showed no detectable optokinetic nystagmus patterns. The visual capability (c) was indicated by the optokinetic gain, the ratio between eye velocity and stimulus velocity. The *prss56* mutants showed a decrease in optokinetic gain for both temporal to nasal (left) and nasal to temporal (right) eye movements, relative to WT. The *fbn1* showed no significant change in optokinetic gain. The number of ETMs/15 s (d) intervals was significantly decreased in both *prss56* and *fbn1* mutants. A baseline spatial frequency (0.15 cycles/degree) and drum velocity (20 degrees/second) were used. See Supplementary Table S2 for all statistics. Error bars: standard error of the mean. Significance: ns = not significant. *P  <  0.05, **P  <  0.01, ***P < 0.001.

The refractive state of the fish was examined by an eccentric photorefractor customized for zebrafish. Owing to the strong reduction of the ocular dimensions in the *prss56* mutants (Fig. 3), it was not possible to perform photorefraction on these eyes. In the *fbn1* mutants, the posteriorly orientated lens subluxation (Fig. 3) resulted in a hyperopic refractive error at 4 and 6 mpf (Fig. 4a), relative to the WT controls. The WT control fish also showed a positive refractive error (Fig. 4a), potentially induced by the small eye retinoscopic artifact.^40^^–^^42^ This hyperopic bias results from the reflection of infrared light from the array on the myelin-rich retinal nerve fiber layer, whereas during normal (emmetropic) conditions light is focused on the photoreceptors. In animals with small eye sizes the neural retina takes up a relatively larger proportion of the total focal length owing to the relatively conserved retinal thickness between species, leading to a hyperopic shift. We, therefore, took the relative differences between WT and mutant fish into account. Additionally, we used the SD-OCT data to compute the relative refractive error^30^ and confirmed the hyperopic shift for both *fbn1* and *prss56* (Supplementary Fig. S4).

The IOP was measured by a custom setup including a rebound tonometer. We found that both *prss56* and *fbn1* mutants showed a significantly increased IOP at 2, 3, 6, and 9 mpf relative to WT control fish (Fig. 4b). For more details on the setup and the protocol, see Figure 1c, Methods, Supplementary Materials, and Supplementary Video File S1.

To assess if the fish retained the ability for basic spatial vision, the OKR was measured in 6 mpf fish. The measurements were performed with a custom OKR setup. See Figure 1d, methods, Supplementary Materials, and Supplementary Video File S2 for more details on the setup and software. For this assessment of basic spatial vision in the mutants, a spatial frequency of 0.15 cycles per degree and drum velocity of 20 degrees per second was used. These settings seemed to be unchallenging for WT fish in previous experiments with this OKR setup.^21^ Our aim was to assess the ability of the fish to correctly respond to basic visual cues rather than performing an extensive assessment of visual acuity. Here, the visual acuity was represented by the optokinetic gain, a measure widely used to depict OKR efficiency computed by the ratio between the slow phase eye tracking velocity and the stimulus velocity.^43^ The optokinetic gain was significantly decreased in the *prss56* mutants, relative to WT controls (Fig. 4c). To more basically quantify the OKR to a stimulus, the number of ETMs^44^^,^^45^ were quantified per 15-second interval. The number of ETMs were significantly reduced in the *prss56* mutants (Fig. 4d) and a small proportion of the *prss56* mutants (*n* = 3) showed no response to the stimulus (Figs. 4c, d). The *fbn1* mutants showed no alteration in the optokinetic gain relative to WT (Fig. 4c), whereas the number of ETMs was significantly decreased (Fig. 4d).

## Discussion

In this study, we present a pipeline that includes the most relevant ocular screening measurements to monitor biometry, refractive status, IOP, and visual capability in zebrafish eyes. Our results demonstrated that zebrafish can be used for in vivo functional screening of genes associated with ocular disorders. In our generated mutant lines (*prss56^re11^* and *fbn1^re12^*), the observed phenotypes showed a close resemblance to the ocular phenotype described in patients with posterior microphthalmia and MFS, respectively, and to previous reported mouse models.^11^^,^^33^^,^^35^^,^^46^ The recognized advantages of zebrafish as an animal model in functional genetics, such as rapid eye development, small size, large clutches of fertilized eggs and the potential for higher throughput assays, together with the evidence and the pipeline provided in our study, highlight the potential of zebrafish for modelling ocular diseases.

The most dramatic biometric change in the *prss56* mutant zebrafish eye is the decrease of the axial length, resembling patients with mutations in *PRSS56* suffering from autosomal recessive nanophthalmos and posterior microphthalmia,^9^^–^^12^ as well as mice with genetic alterations in *Prss56*.^11^^,^^33^ We diagnosed the condition of the *prss56* mutants as nanophthalmos because of the significant decrease in both the anterior and posterior segments of the eye in combination with an elevated IOP. While performing SD-OCT studies (image acquisition and segmentation), it was evident that the prss56 mutant showed a decrease in the vitreous chamber; for that reason, examiners were not masked to the genotype. However, image acquisition and manual segmentation were performed independently. Furthermore, owing to the severity of the phenotype in the *prss56* zebrafish mutants, we could not measure refractive error; however, the decrease in the vitreous chamber depth of the fish will result in a strong hyperopic refractive error as reported in patients with autosomal recessive posterior microphthalmia as well as the *Prss56* KO mice.^9^^,^^13^^,^^33^^,^^47^^,^^48^ This finding is further supported by the hyperopic shift observed when the SD-OCT data were used to calculate the relative refractive error.

In vertebrate species, the retinal thickness is related inversely to the globe volume and, therefore, decreases over time.^36^^–^^39^ Consequently, the retinal thickness of the nanophthalmic *prss56* fish mutants remained relatively thicker over time, whereas the retinal thickness of WT zebrafish rapidly decreased between 2 and 6 mpf. In addition to the *prss56* fish mutant, the nanophthalmic *Prss56* mouse mutants also showed a relatively thicker retina.^33^

The *prss56* mutant fish showed a decreased lens thickness along with the total ocular volume, whereas the lens of the mouse mutants did not change relative to controls.^11^^,^^33^ In patients with posterior microphthalmia, thicker lenses have been reported; however, these lenses were measured relatively to the globe volume.^9^^,^^10^ The decrease in lens diameter in the *prss56* mutant fish may be related to the severe decrease in the anterior and posterior segments. This decrease may have led to a decreased lens circulation, because aqueous humor outflow has also been significantly compromised in the nanophthalmic *Prss56* KO mice.^34^ Although not tested in this study, the potential negative effect on lenticular circulation may have ultimately led to impaired development or degeneration of lens fibers in the zebrafish mutants.

The *prss56* fish mutants showed a significantly elevated IOP. In humans, an elevated IOP has been reported in people with pathogenic genetic variants in *PRSS56*^11^^,^^13^^,^^34^ and in mice with mutations in *Prss56*.^11^ The loss of *prss56* in zebrafish also leads to a decreased visual acuity based on the optokinetic gain and number of ETMs per interval coherent to the reduced visual acuity found in patients with mutations in *PRSS56*.^12^

In the *fbn1* mutant fish, the most distinctive ocular phenotype observed was a subluxation of the lens (ectopia lentis), characterized by the increased anterior chamber depth. In WT conditions the assessment of anterior chamber depth in zebrafish is challenging because the lens is in contact with the back of the cornea. In those cases, calculation of the anterior chamber volume might be a complementary metric. In the *fbn1* mutant, on the contrary, a evident large anterior chamber depth was observed as results of the subluxation of the lens. Parallel, in patients with MFS, ectopia lentis is considered a cardinal feature, and myopia (>3 diopters) is one of the systemic features included in the score used for diagnosis.^35^^,^^49^ Other ocular manifestations include increased axial length, flattened corneas and hypoplastic iris or ciliary muscle.^16^^,^^17^^,^^50^^–^^52^ Myopia is one of the systemic features used in the diagnosis of MFS. However, assessing the refractive state of the eye of patients with MFS might be challenging as refraction depends on various parameters, such as axial length, corneal curvature, and lens optics. Increased axial length will result in myopia, flatter corneas will lead to hyperopia, and ectopia lentis can induce myopia, high astigmatism (in cases of partial lens subluxation), or high hyperopia (in cases of complete lens subluxation).^16^^,^^17^^,^^50^^,^^53^ In the *fbn1* mutant, we found a strong hyperopic shift in the refractive status of the eye, but no changes in the axial length. The hyperopic shift observed in the *fbn1* mutant fish is explained by two factors: (1) in zebrafish, the spherical lens is responsible for the full optical power of the eye (the refractive index of the cornea and water is nearly identical, and thus corneal curvature does not contribute to refraction), and (2) the posterior subluxation of the lens caused the optical field to shift backwards. Other reported ocular manifestations of MFS include cataract, glaucoma, and retinal detachment.^14^^,^^16^^,^^17^^,^^50^ Cataract has been reported in mouse models of MFS,^35^ yet in this study we have not observed opacifications in the lenses of the *fbn1* zebrafish mutants. Although the induced mutation in the *fbn1^re12^* is predicted to lead to a premature stop codon and thus truncated protein, we cannot rule out that the phenotypic differences observed in the *fbn1^re12^*, that is, the lack of axial elongation and cataract development, might be explained by a (partially) remaining functionality of the truncated protein product.

We showed that the *fbn1* zebrafish mutants developed an elevated IOP in the juvenile stage (2 mpf) that persisted into late adulthood (9 mpf). Similarly, patients with MFS and Weill–Marchesani type 2 syndrome with mutations in *FBN1* often develop elevated IOP as a result of glaucoma.^14^^,^^15^^,^^50^^,^^54^^,^^55^ In contrast with the *fbn1* zebrafish mutants, the *Fbn1* mouse mutants did not show a significant increase in IOP, except for two animals suffering from strongly cataractous lenses.^35^ Finally, the *fbn1* fish mutants showed a decreased number of ETMs per 15-second interval, indicating a decrease in spatial vision capability, similar to MFS patients.^53^^,^^56^

To standardize our ocular phenotype pipeline, we generated KO fish for two genes (*prss56* and *fbn1*) involved in Mendelian diseases with ocular manifestations; furthermore, single nucleotide polymorphisms in these genes are associated with refractive error in the general population.^1^^,^^2^ This pipeline can be used to study a variety of ocular phenotypes in the zebrafish with emphasis to the study of refractive error. Therefore, ocular biometry (studied with SD-OCT) is considered an essential outcome measure. Moreover, we show that SD-OCT also provides other relevant information, including lens opacity, and position. Both parameters are useful in the study of cataracts or when lens subluxation is suspected. Furthermore, we demonstrate that eccentric photorefraction is a complementary assessment to the ocular biometry. Rather than focusing on the diopter values per se, we focused on the differences in photorefraction between WT and mutant fish. It is important to note that, in cases of severe refractive errors, as observed in the nanophthalmic eye of the *prss56* mutant, eccentric photorefraction is not possible; we have also observed this phenomenon in the highly myopic *lrp2* (bugeye) mutant.^30^ In those cases, calculating the relative refractive error from the SD-OCT data is an alternative. Our pipeline also shows the potential use of rebound tonometry to study IOP. Rebound tonometry has been used in rodents,^57^ chicken,^58^ and KOI carp^59^ to study IOP, however; this technique has not been used before in zebrafish. Finally, our pipeline assesses the visual capability of zebrafish using OKR. Our OKR assessment focuses on a single velocity and spatial frequency and is used to illustrate a general indication of the basic visual capability of the fish. However, the same setup and software described in this article can be used for more comprehensive assessments of visual acuity, as is also described in previous articles.^60^^,^^61^

With respect to our examples of functional genetics in zebrafish, we found an inhibitory effect on axial growth in our *prss56* zebrafish mutants,^9^^–^^12^^,^^33^ similar to the posterior microphthalmos and nanophthalmos in patients. In addition, we found that these mutants showed an elevated IOP and decreased visual acuity, which have also been described in mouse models and patients.^9^^–^^12^^,^^33^ In contrast, the *fbn1* zebrafish mutants did not show an increase in axial length, one of the hallmarks of MFS and Weill–Marchesani syndrome patients and the *Fbn1* mouse mutants.^14^^–^^17^^,^^35^^,^^62^ These mutants did, however, have other typical ocular manifestations associated with mammalian *FBN1* mutations, that is, ectopia lentis, an elevated IOP, and a decreased visual performance.

In summary, we present a series of tests that can be used in vivo to study ocular phenotypes in zebrafish. Using SD-OCT, we successfully measured biometric changes in both mutant lines and with eccentric photorefraction we detected, in the in *fbn1^re12^* mutant, hyperopia induced by the posterior shift of the lens. IOP measurements in zebrafish have previously been performed by servo-null electrophysiology,^63^ an invasive method using glass microelectrodes to penetrate the eye. Here, we demonstrate a faster and noninvasive method for measuring IOP in zebrafish. This pipeline can be used in future studies, such as the development of intervention strategies for myopia, for example, the screening of pharmacological compounds that inhibit ocular elongation, the effects of environmental modifications, or functional studies into the effects of specific genetic variants.

## Supplementary Material

Supplement 1

Supplement 2

Supplement 3

Supplement 4
