# Supplementary material for: Zebrafish: An In Vivo Screening Model to Study Ocular Phenotypes
Source: Transl Vis Sci Technol. 2022 Mar 14;11(3):17. doi: 10.1167/tvst.11.3.17 (PMC8934544; doi:10.1167/tvst.11.3.17)
Supplement: Supplement 1 [file tvst-11-3-17_s001.pdf]

Supplementary Materials

Zebrafish: an in vivo screening model to study ocular phenotypes

Expression of *prss56* and *fbn1* in the zebrafish eye 2

Protocol # 1 Spectral-domain optical coherence tomography in zebrafish 3

Protocol # 2 Eccentric photorefraction in zebrafish 6

Protocol # 3: Tonometry 8

Protocol # 4: Optokinetic response measurements in zebrafish 10

Supplementary Tables

Supplementary Table S1. List of primers used 13

Supplementary Table S3. Protein conservation 13

Supplementary Figures

Supplementary Figure S1. Biometrical changes were independent of body length 14

Supplementary Figure S2. Phylogeny of vertebrate PRSS56 14

Supplementary Figure S3. Transcript analysis for prss56 15

Supplementary Figure S4. Relative refractive error 16

Protein conservation and expression of *prss56* and *fbn1* in the zebrafish eye

We evaluated the protein conservation between zebrafish and human. Zebrafish Prss56 protein appeared to show only a mild conservation (29.6% overall identity) with human PRSS56, whereas human FBN1 protein aligned well with zebrafish Fbn1 (66.3% overall identity, Supplementary Table S3). Nevertheless, the serine protease domain of Prss56, retained a stronger conservation (58% identity) with the human homologue (Supplementary Table S3). The phylogenetic tree (Supplementary Fig. S2) shows that after the branch point of the vertebrate lineage into the fish and mammalian lineage, zebrafish *prss56* has a relatively early separation from the rest of the fish and teleost lineage. Whereas many teleost fish obtained one or more copies of *prss56* during genome duplication events, zebrafish *prss56* retained a single copy (Supplementary Fig. S2).

As NCBI predicted two isoforms of zebrafish *prss56* (Fig. 2a), we developed 4 primer sets (Supplementary Fig. S3a, Supplementary Table S1) spanning the entire cDNA region of *prss56* to test whether the two isoforms were expressed in the zebrafish eye. When comparing the two predicted isoforms, we found an identity score of 97.3% (Supplementary Table S3), and a 91bp gap located in the 5' UTR of XM_017352214.2 (short isoform) relative to XM_017352217.2 (long isoform). All 4 primer sets generated amplicons according to their expected product size (Sup. Fig. 3b). Primer set 1 included the region of the 5'UTR (Supplementary Fig. S3a), and as expected, it amplified two different products as shown in Supplementary Fig. S3c. We sequenced both products individually and aligned them to the predicted NCBI sequences of both isoforms (Supplementary Fig. S3c-e). We found the 91bp region of the 5'UTR in the longer fragment (99% identity), whereas absent from the shorter fragment (Supplementary Table S1). The differences between both isoforms were limited to the 5'UTR region and are, therefore, not expected to result in structural and functional differences for the Prss56 protein.

Protocol # 1: Spectral-domain optical coherence tomography in zebrafish

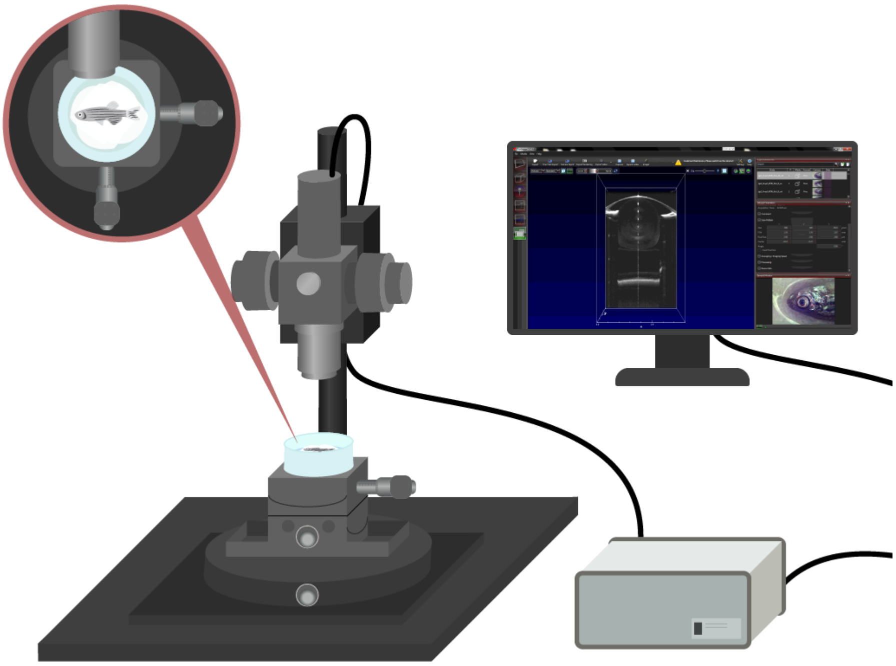

Materials Needed

- PC
- SD-OCT device (Thorlabs SD-OCT 900 nm Ganymade system, 930nm)
- Long-Depth-of-Focus Scan Lens Kit, LSM04.
- Dual-Axis Goniometer (GNL20, Thorlabs)
- 0.016% tricaine methanesulfonate solution (MS222, Sigma Aldrich), buffered to pH = 7
- Caliper for body length measurements (resolution 0.001mm).
- Tissue dish (60x15mm)
- Foam rubber
- 10ml syringe

Preparation

Preselect fish; the body length needs to be equal (max 1% variation) between groups. Body length is measured by a caliper from the tip of the head to the proximal end of the caudal fin.

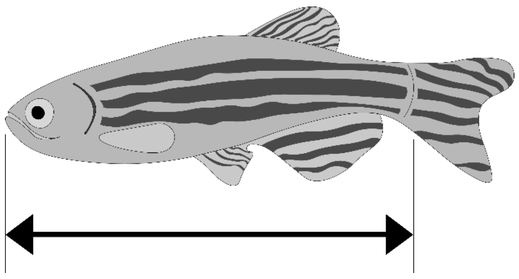

Fish handling before imaging

Before imaging, each fish needs to be placed in a tank with buffered (pH=7) 0.016% tricaine methanesulfonate solution (MS222, Sigma Aldrich), for at least 2 minutes. Gill movement should be tracked and minimized during measurements to prevent breathing artifacts in the SD-OCT scan. Increase the tricaine concentration if needed. Once anesthetized, fish can be placed gently on the foam of the tissue dish.

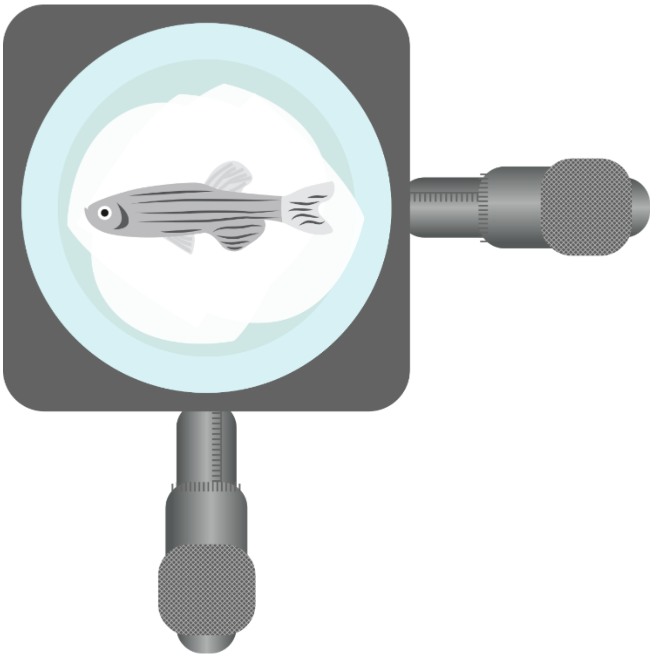

If the fish shows increased gill movement during measurements, a droplet of extra undiluted (0.16%) tricaine solution can be applied directly onto the gills to rapidly anesthetize the fish. Keep the spatial positioning of each fish constant between measurements. Make sure the fish stays wet during the preparations.

Imaging /Scanning

For each measurement, keep the same orientation of the fish. Position the fish so that the eye is perpendicular to the lens and one side of the head is covered in tricaine solution and the other half, with the eye facing upwards to the scanner, is not. Adjust the angle of the dual-axis goniometer till in the mid plain of both the x and the y view: 1) a reflection (artifact) is visible that indicates that the highest point of the cornea is perpendicular to the IR light 2) the iris is positioned horizontally and perpendicular to the IR light.

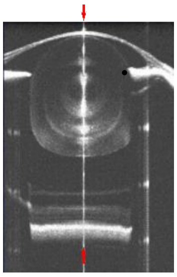

Take a 3D recording of the fish eye that can be further analyzed in MATLAB. The following field of view settings can be used for 2 and 4 mpf fish in the case of the Thorlabs 900nm Ganymade system and LSM04 lens kit:

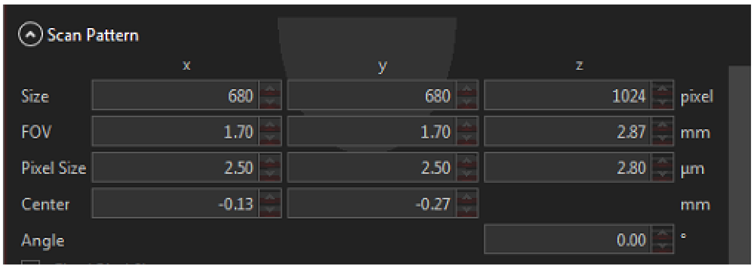

Fish handling after imaging

After imaging, fish need to recover from the anesthesia in aquarium water. Recovery of the gill movement needs to be monitored, if gill movement does not increase after 1 minute, a syringe with system water can be used to gently flush the gills.

Data analysis in MATLAB

OCT files are unzipped and pre-processed using custom MATLAB script (available on request). *Z*, *x*, and *y* views can be displayed adjacently. The *y* view represents the individual B scans and is not sensitive to breathing artifacts. Therefore, this view is used to annotate the ocular compartments. Brightness settings can be adjusted. The crosses are placed on the boundaries of the ocular components and can be freely placed along the horizontal axis as only the vertical distance to the previous boundary is used to calculate the distance and compute the axial length. Axial length is calculated from the anterior part of the cornea to the anterior boundary of the RPE as the posterior border of the RPE is faded.

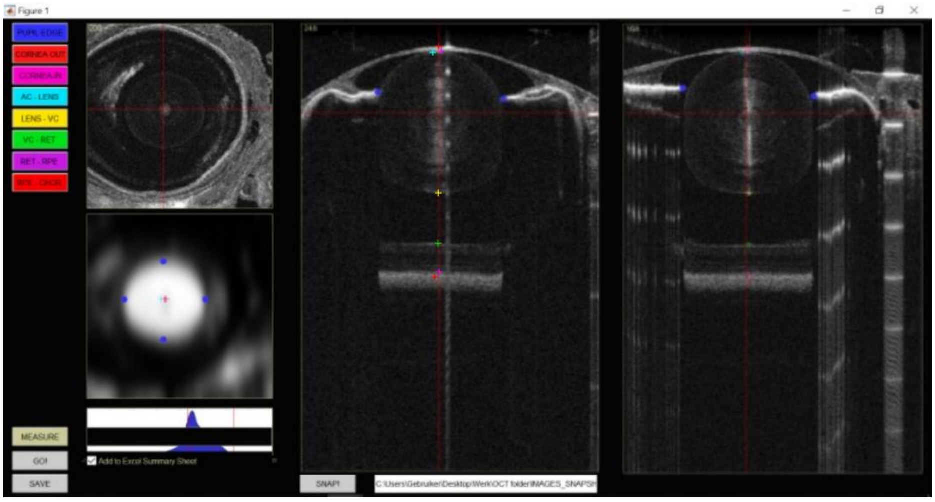

The dimensions of the ocular components were corrected for the specific refractive index of each ocular component:

- The cornea 1.33 [1–3]
- The lens (gradient refractive index) 1.40 [1–6]
- The anterior and vitreous chamber 1.34 [3,7,8]
- The retina 1.38 [3,7,8]

Protocol # 2: Eccentric photorefraction in zebrafish.

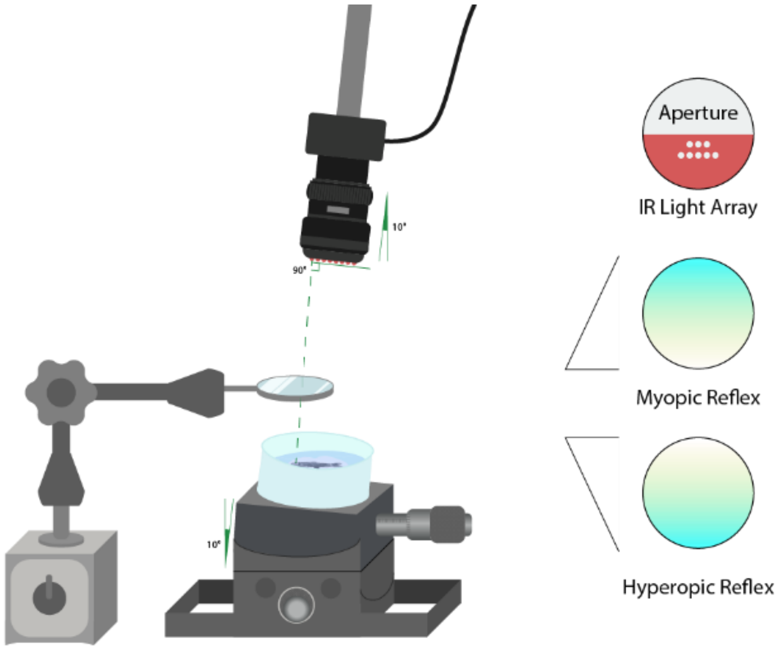

Materials Needed

- PC
- Custom software (available on request)
- USB Camera
- Lens (Ricoh, TV lens 50mm 1:1,4)
- 850 nm long-pass filter (Midopt, LP695-46).
- Lens cap with array of infrared-emitting diodes
- Adjustable camera mount, positioned at 10 degrees relative to water surface
- Dual-Axis Goniometer (GNL20, Thorlabs), positioned at 10 degrees relative to water surface
- Electrical motor (Makeblock, 37MM/DC12,0V/50RPM±12%/ 1:90)
- Rotating drum
- Tissue dish (60x15mm)
- Foam rubber
- 0.016% tricaine methanesulfonate solution (MS222, Sigma Aldrich), buffered to pH = 7
- 10ml syringe

Fish handling before measurements

Before imaging, each fish needs to be placed in a tank with buffered (pH=7) 0.016% tricaine methanesulfonate solution (MS222, Sigma Aldrich), for at least 2 minutes. Gill movement should be tracked and minimized during measurements to prevent disruption of measurements. Fish need to be placed in a container with fresh system water and fixed gently on the foam rubber. Fixation was accomplished by spanning a rubber band (.i.e., water proof tape) over the foam and sliding the fish gently under the band. If the fish starts moving or gill movement increases during measurements, increase the tricaine concentration in the container. Both the camera and fish should be slightly angled (<10 degrees) relative to the water surface to prevent the reflection of the light array on the water surface.

Measurements

Custom C++ software is used to measure the gradient of refraction in real-time by placing the circle over the pupil. The size of the circle can be altered to fit the pupil size. Right mouse button saves each measurement.

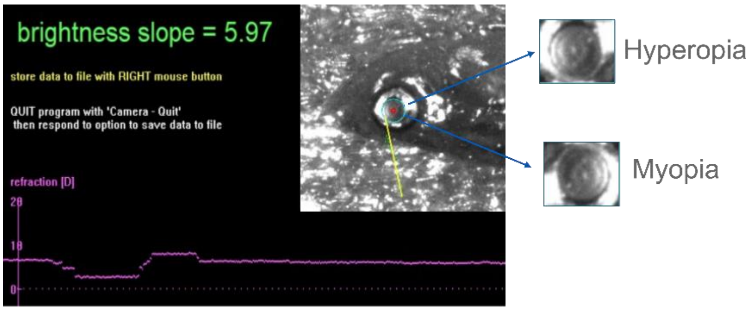

For each eye multiple subsequent measurements are taken and averaged.

Calibration

The slope of the brightness gradient was converted into RE by calibrating the system with ophthalmic lenses in the −5 to +10D range. When plotted, this should show a high coefficient of determination (r2 = 0.971). With our specific setup, we found a conversion factor of 1.924. The conversion factor was used to convert the slope of the brightness profile into refractive status in Diopter.

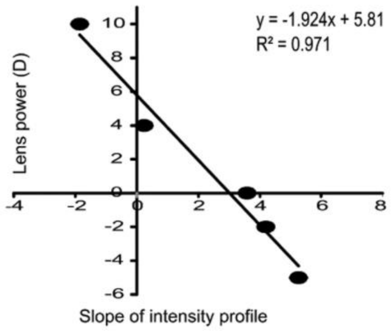

Fish handling after measurements

After imaging fish need to recover from the anesthesia in aquarium water. Recovery of the gill movement needs to be monitored. If gill movement is not recovered after 1 minute, a syringe with fresh system water can be used to gently flush the gills.

Protocol # 3: Tonometry

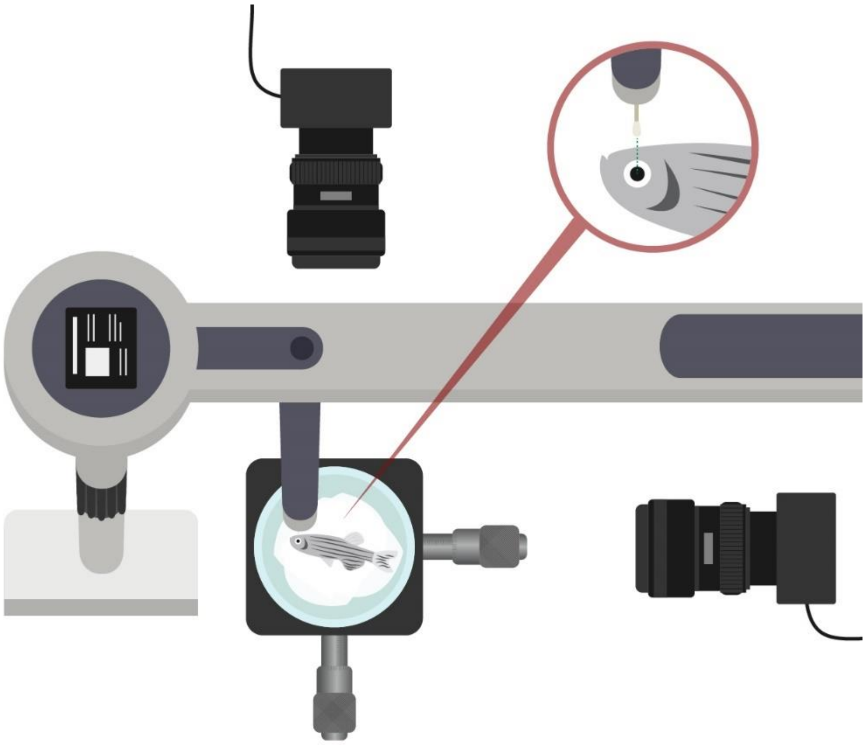

Materials Needed

- iCare Pro rebound tonometer
- Movable stage
- PC
- 2x USB Camera
- 2x Lens (Ricoh, TV lens 50mm 1:1,4)
- 2x Adjustable camera mount
- Dual-Axis Goniometer (GNL20, Thorlabs)
- Tissue dish (60x15mm)
- Foam rubber
- 0.016% tricaine methanesulfonate solution (MS222, Sigma Aldrich), buffered to pH = 7
- 10ml syringe

Preparation

Build the setup as illustrated above. The two cameras are used to monitor the position of the eye relative to the probe from two angles. The iCare Pro is fixed to a stand/movable stage. A tissue dish with a foam rubber attached to a metal ring is used to make sure the fish stays wet as well as anesthetized during measurements.

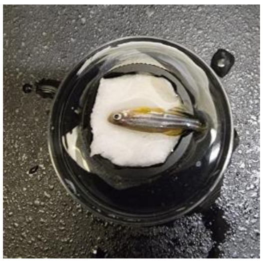

Fish handling before measurements

Before imaging, each fish needs to be placed in a tank with 200ml of buffered (pH=7) 0.016% tricaine methanesulfonate solution (MS222, Sigma Aldrich), for at least 2 minutes. Gill movement should be tracked and minimized during measurements to prevent disruption of measurements. Fish need to be placed on a foam rubber. The center of the fish cornea is positioned perpendicular to the probe by adjusting the goniometer. Make sure the fish stays wet continuously during these preparations. The eye that is measured should be positioned (just) outside the water surface.

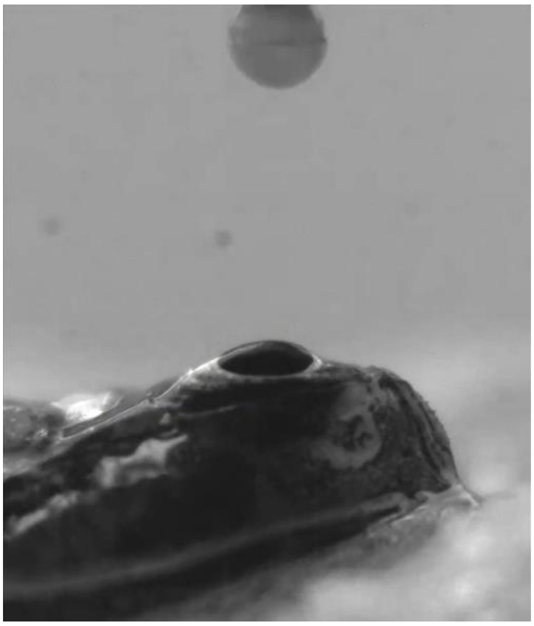

Measurements

See Supplementary Video S1 for the probe movement synchronized with the display of the IOP on the tonometer. 6 measurements are averaged by the iCare Pro.

Fish handling after measurements

After imaging fish need to recover from the anesthesia in aquarium water. Recovery of the gill movement needs to be monitored. When the gill movement is not recovered after 1 minute a syringe with water can be used to gently flush the gills.

Protocol # 4: Optokinetic response measurements in zebrafish

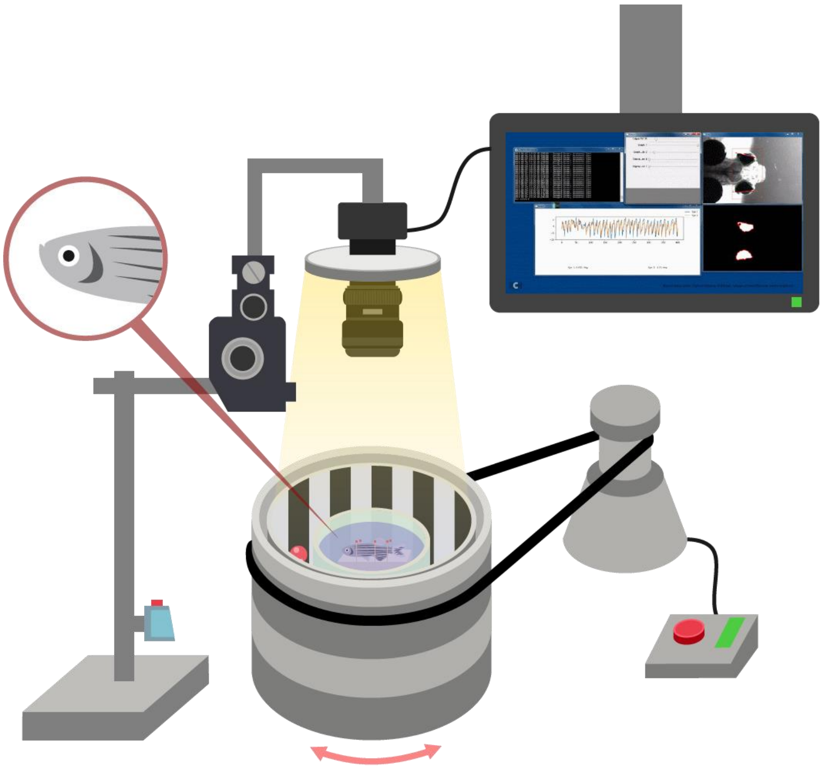

Materials Needed

- PC
- Custom eye tracking software (available on request)
- Analysis software (available on request)
- Backlight (TCAM, Ring Light, 0-100%/12V/6000-7000K)
- Infrared-emitting diode (XIASONGXIN LIGHT, 9-12V/10W/1050MA)
- USB Camera
- Lens (Ricoh, TV lens 50mm 1:1,4)
- 850 nm long-pass filter (Midopt, LP695-46).
- Tachometer (Autoleader, NJK-5002C)
- Electrical motor (Makeblock, 37MM/DC12,0V/50RPM±12%/ 1:90)
- Rotating drum
- 0.016% tricaine methanesulfonate solution (MS222, Sigma Aldrich), buffered to pH = 7

Preparation

Build setup as shown above including:

1. A camera
2. A ring light for drum illumination (visible light)
3. A long pass filter attached to camera lens
4. An infrared LED illuminator to illuminate the fish head from below
5. A motor to control drum movement
6. A power regulator to control the motor velocity

7. A tachometer detecting a magnet on the drum to measure the velocity
8. A cylindrical transparent fish aquarium with a fixation platform consisting of a foam rubber with pins to fixate the fish.
9. A drum
10. Paper with an alternating black and white pattern with the desired contrast and spatial frequency.

Fish handling before measurements

Before imaging, each fish needs to be placed in a tank with buffered (pH=7) 0.016% tricaine methanesulfonate solution (MS222, Sigma Aldrich), for at least 2 minutes. When the fish is anesthetized place the fish ventrally on the fixation platform and fixate by inserting pins into the foam, gently clamping the fish. Fill the tank with fresh aquarium water and let the fish fully recover for at least 5 minutes. Insert a spatial frequency and contrast level of choice. Set the right drum speed by adjusting the power to the motor. Switch on the infrared illuminator to properly visualize the eyes from below.

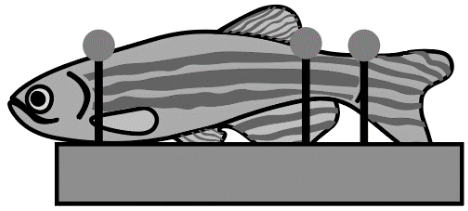

Measurements

Custom software is used for live eye tracking.

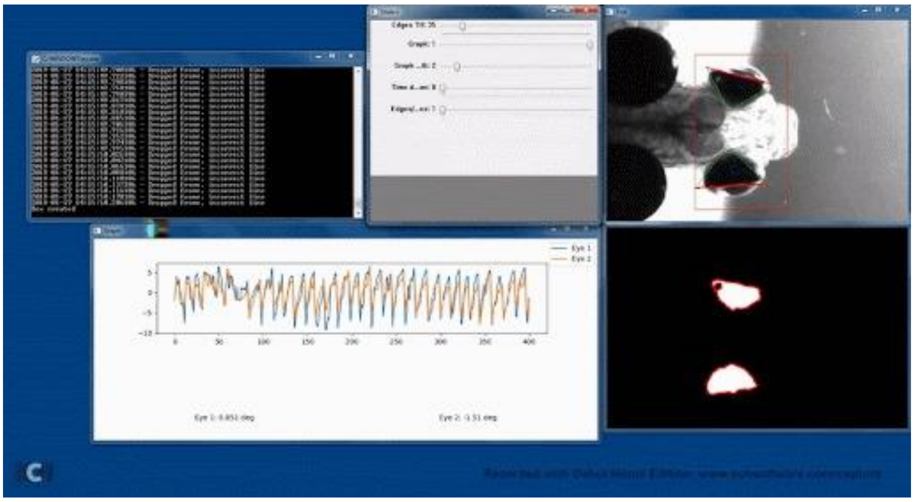

Click two times on the live video to indicate where the software should position the eye overly. Adjust the eye tracking settings till the red rectangle and two red triangles are positioned correctly on the eyes. The OKN pattern is visualized in the lower left screen

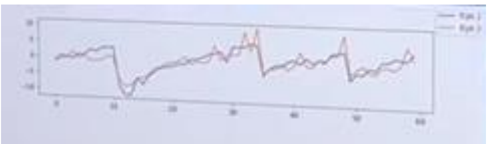

For each measurement, the fish need to be pre-stimulated for 5 seconds in both directions. After a 30 second break the measurement can start. Repeat till the fish performed uninterrupted OKN movements for 20 seconds. See Supplementary Video File S2 for live video of the eye tracking.

Data analysis

A second custom script is used to analyze the data. The software automatically detects the slopes of the slow pursuits that need to be analyzed. However, the selection can be altered manually as well. The software writes the data into an excel file that can be used for further analysis.

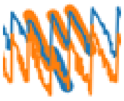

The slope represents the eye velocity during this trajectory of the OKN. The optokinetic gain can be computed by taking the ratio of the eye velocity of the slow pursuit and the drum velocity.

Supplementary Tables

Supplementary Table S1.

Name	Sequence	Amplicon size (bp)
***prss56* seq 1 FW**	GCACAGCTTCTGTCACTCAC	969/877
***prss56* seq 1 RV**	TGGGTTAAACTTCGGGTGGG	
***prss56* seq 2 FW**	CGCAGTGAGAGTTACTGGACTG	932
***prss56* seq 2 RV**	CCTAGCTGCTCTGTTTCAAGTG	
***prss56* seq 3 FW**	CGCCTCGCTCTTGACAAATG	976
***prss56* seq 3 RV**	ACTTCAGATCAACCTGCACCTC	
***prss56* seq 4 FW**	TGAGGAGGAGAAAGAGAGACTC	576
***prss56* seq 4 RV**	TGAAGATCAACCTCTGCCCTTG	
***fbn1* FW**	TCAACTCCACCGAGTACAAG	497
***fbn1* RV**	AGCGAATTCATTGGTGCTGG	

Supplementary Table S3.

Sequence input 1	Sequence input 2	Identity score (%)
Human PRSS56<br>NP_001356777.1	Zebrafish Prss56 protein<br>XP_017207706.2	30
Human serine protease domain<br>NP_001356777.1; aa105-335	Zebrafish serine protease domain<br>XP_017207706.2; aa191-419	58
Longer transcript of zebrafish *prss56*<br>XM_017352217.2	Shorter transcript of zebrafish *prss56*<br>XM_017352214.2	97
Predicted 5'UTR gap sequence of zebrafish *prss56*	Sequenced cDNA shorter isoform of zebrafish *prss56*	0
Predicted 5'UTR gap sequence of zebrafish *prss56*	Sequenced cDNA longer isoform of zebrafish *prss56*	99
Human FBN1<br>NP_000129.3	Zebrafish Fbn1<br>XP_017207479.2	66

Supplementary Figures

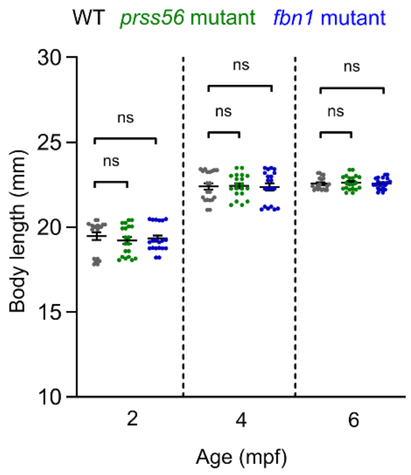

Supplementary Figure S1. Biometrical changes were independent of body length. Body length measurements of *prss56*^{re11} and *fbn1*^{re12} mutants (rounded). Body length was measured from the tip of the head to the proximal end of the caudal fin. During SD-OCT measurements, both fish lines were compared with size-matched WT controls. Sample size: n=10 fish for n=20 eyes. Error bars: SEM. Significance: ns = not significant, *$p < 0.05$, **$p < 0.01$, ***$p < 0.001$. Mpf: months post-fertilization.

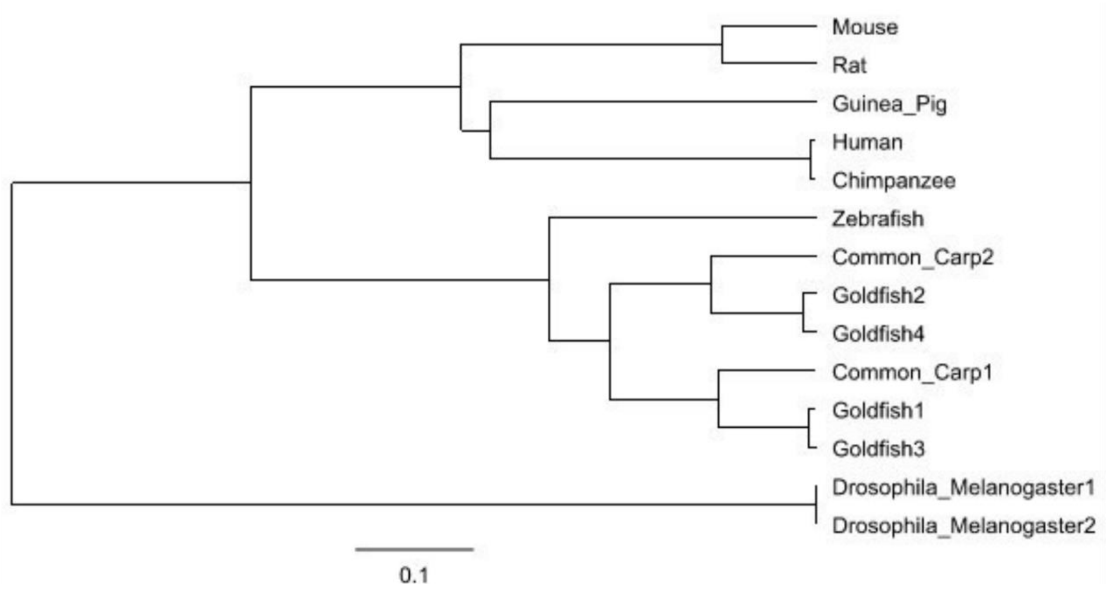

Supplementary Figure S2. Phylogeny of vertebrate *PRSS56*. Phylogenetic tree showing the distribution of vertebrate *PRSS56*. Zebrafish *prss56* has an early separation from the rest of the teleost lineage retaining a single copy.

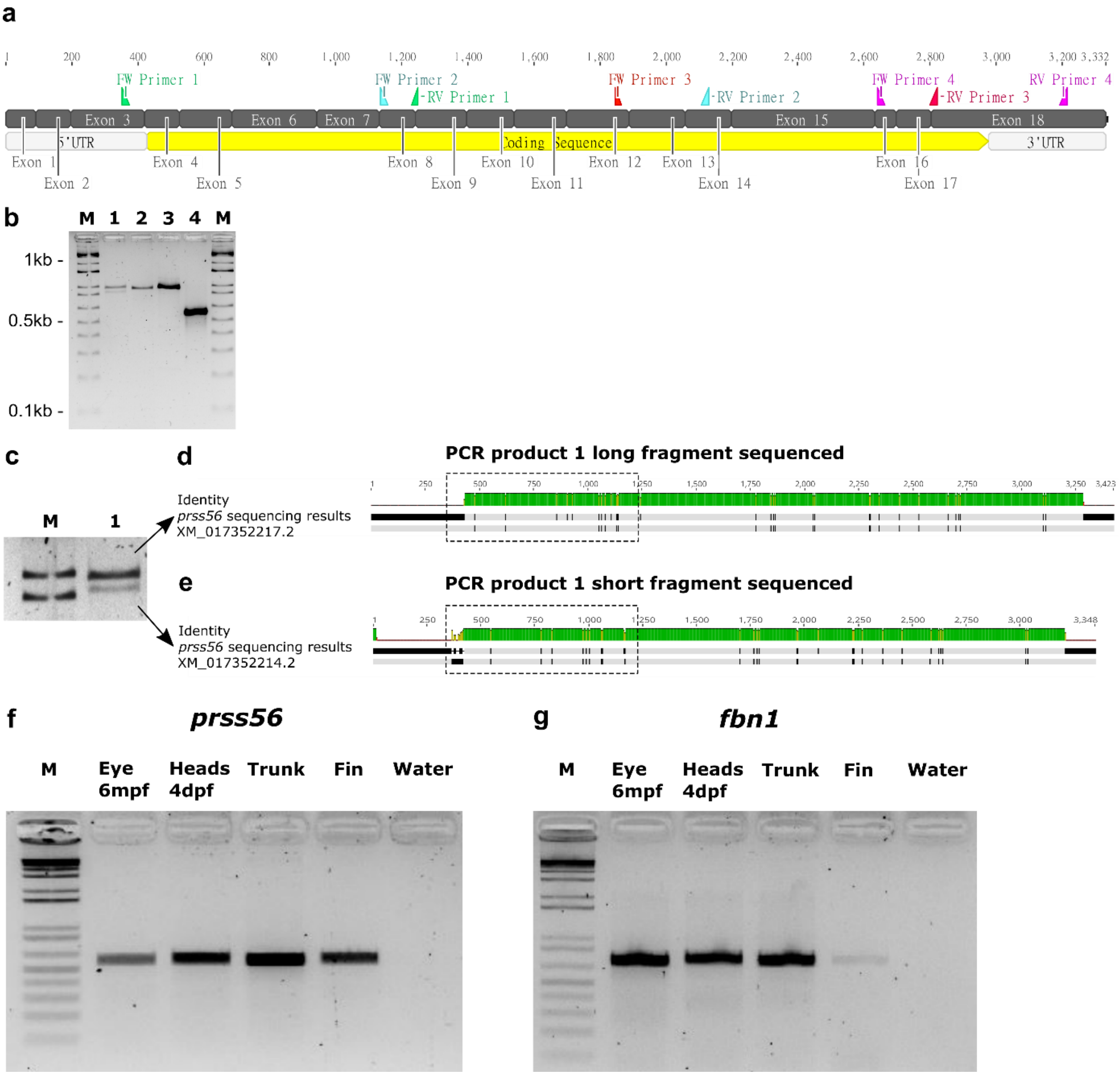

Supplementary Figure S3. Transcript analysis for *prss56* and primer validation. Isoform determination in adult (6mpf) zebrafish eyes. **a** Four primer sets spanning the full *prss56* coding DNA sequence were designed. **b** PCR products are shown on agarose gel, each PCR product is represented by a number. **c** PCR product 1 showed two bands. **d, e** Both products were separately amplified, sequenced, and aligned to the reference sequences XM_017352214.2 (shorter isoform) and XM_017352217.2 (longer isoform). **f**, **g** Agarose gel loaded with RT-PCR product of isolated RNA extracted from respectively 6mpf zebrafish eyes, 4dpf heads, and the trunk and caudal fin of 6mpf fish. Both *prss56* (**f**) and *fbn1* (**g**) were expressed in these tissues. For *prss56* primer set 4 was used. See Supplementary Table S1 for primer sequences. Dpf: days post-fertilization, mpf: months post-fertilization, M: marker.

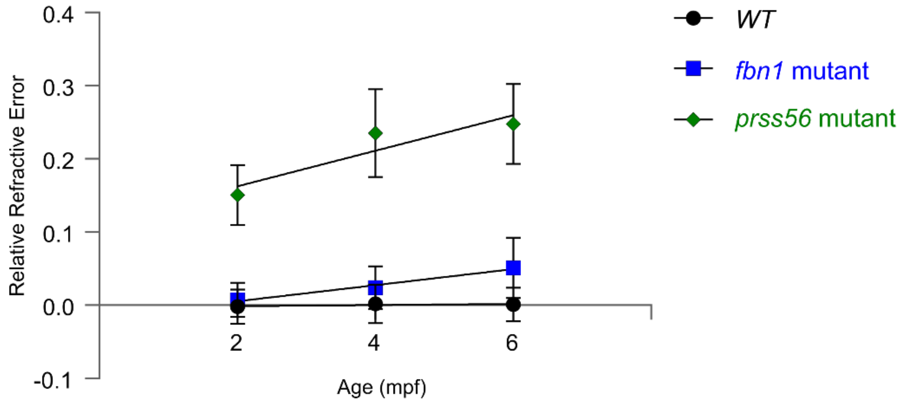

Supplementary Figure S4. Relative refractive error

Relative refractive error calculated for *prss56*^{re11} and *fbn1*^{re12} mutants. Error bars: SE. Mpf: months post-fertilization

References

1. Greiling TMS, Clark JI. The transparent lens and cornea in the mouse and zebra fish eye. *Semin Cell Dev Biol*. 2008;19(2):94-99.

2. Verma Y, Divakar Rao K, Gupta PK. In-vivo imaging Of adult zebrafish using optical coherence tomography. In: *1st Canterbury Workshop on Optical Coherence Tomography and Adaptive Optics*. SPIE; 2008. doi:10.1117/12.814914

3. Rao D, Verma Y, Patel H, Gupta P. Non-invasive ophthalmic imaging of adult zebrafish eye using optical coherence tomography. *Curr Sci*. 2006;90.

4. Hoshino M, Uesugi K, Yagi N, Mohri S, Regini J, Pierscionek B. Optical properties of in situ eye lenses measured with X-ray Talbot interferometry: a novel measure of growth processes. *PLoS One*. 2011;6(9):e25140.

5. Verma Y, Rao KD, Suresh MK, Patel HS, Gupta PK. Measurement of gradient refractive index profile of crystalline lens of fisheye in vivo using optical coherence tomography. *Appl Phys B*. 2007;87(4):607-610.

6. Young LK, Jarrin M, Saunter CD, Quinlan RA, Girkin JM. Non-invasive in vivo quantification of the developing optical properties and graded index of the embryonic eye lens using SPIM. *Biomed Opt Express*. 2018;9(5):2176-2188.

7. Drexler W. Optical coherence tomography: Technology and applications. In: *2013 Conference on Lasers & Electro-Optics Europe & International Quantum Electronics Conference CLEO EUROPE/IQEC*. IEEE; 2013. doi:10.1109/cleoe-iqec.2013.6802013

8. Hariri S, Moayed AA, Dracopoulos A, Hyun C, Boyd S, Bizheva K. Limiting factors to the OCT axial resolution for in-vivo imaging of human and rodent retina in the 1060 nm wavelength range. *Opt Express*. 2009;17(26):24304-24316.
